# A Singleton Infant with Bilateral Renal Agenesis and Normal Pulmonary Function

**DOI:** 10.1155/2017/1710371

**Published:** 2017-11-27

**Authors:** Lovya George, Winston Manimtim, Jotishna Sharma

**Affiliations:** Division of Neonatology, Children's Mercy Kansas City, 2401 Gillham Road, Kansas City, MO 64108, USA

## Abstract

Bilateral renal agenesis leads to anhydramnios and other sequelae including pulmonary hypoplasia. There have been rare case reports of normal pulmonary function in the presence of bilateral renal agenesis in monoamniotic discordant twins, but this has never been reported in a singleton pregnancy. The few reported cases in twins have all been fatal in the neonatal period with no reported cases of survival beyond 2 months. We describe the first case of a singleton infant with bilateral renal agenesis who had normal pulmonary function and did well on peritoneal dialysis for 4 years while awaiting a renal transplant.

## 1. Introduction

Absence of fetal urine production is known to result in oligohydramnios and anhydramnios [1], causing fetal malformations described in Potter sequence including flattened facies, limb malformations, low set abnormal ears, and pulmonary hypoplasia. As the presence of amniotic fluid is vital to lung development, bilateral renal agenesis is deemed to be uniformly fatal due to associated pulmonary hypoplasia. If this condition is detected prenatally, parental counselling is almost universally directed towards comfort care and avoidance of heroic measures after birth. We present a case of an infant girl with prenatally unknown bilateral renal agenesis, which was diagnosed after birth and had normal pulmonary function.

## 2. Case

**Figure 1:**
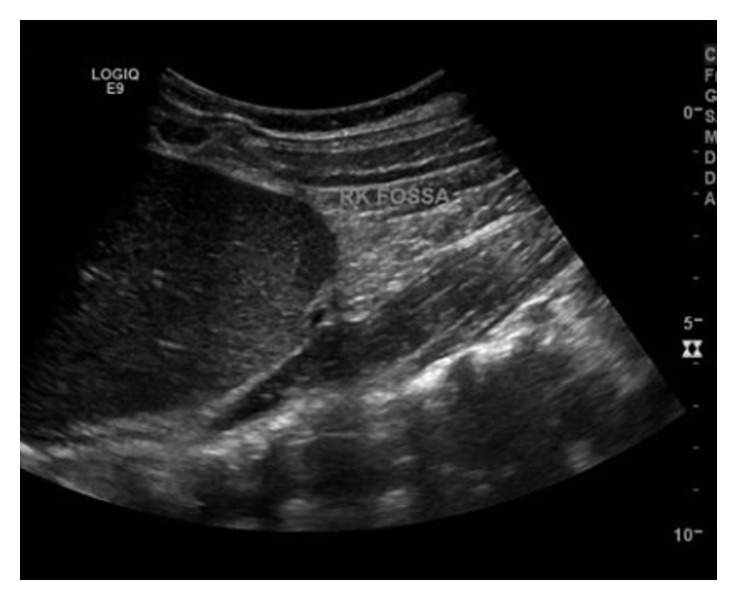
Long-axis/sagittal ultrasound image from the right upper quadrant showing no evidence of renal parenchyma.

**Figure 2:**
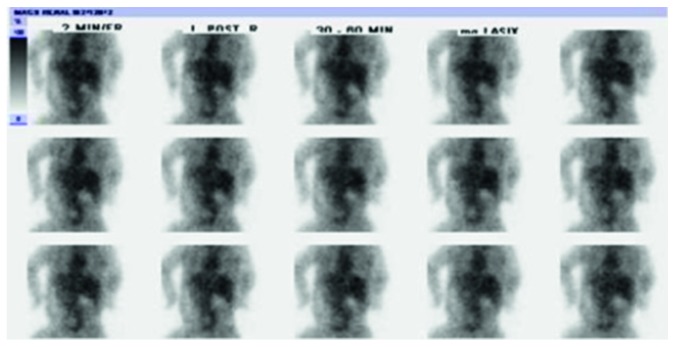
Sequential posterior planar images from MAG3 scan showing no perceptible renal activity. A large amount of other soft tissue activity/blood pool activity is present.

**Figure 3:**
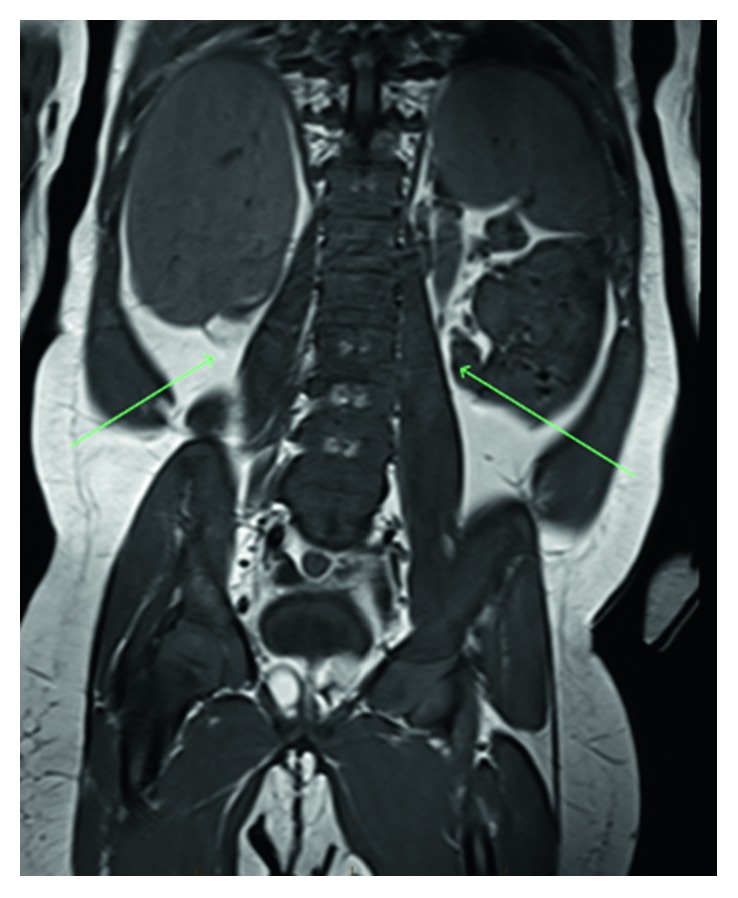
Coronal T1-weighted MRI image showing absence of renal tissue (arrows).

A term female infant born at 39 weeks by repeat caesarean section to a 31-year-old gravida 3, para 3 mother after uncomplicated pregnancy was transferred to our Neonatal Intensive Care Unit (NICU) at approximately forty-eight hours of life due to history of no urine output, ambiguous genitalia, and imperforate anus. Apgar scores were 7, 7, and 9 at 1, 5, and 10 minutes, respectively, with birth weight of 2.9 kg. Physical examination did not reveal any facial dysmorphism. Significant findings on clinical exam included an abnormal genitourinary exam with hypoplastic labia majora, small fused labia minora, and a single opening in the urethral/vaginal area which yielded turbid white fluid when a catheter was placed temporarily. Constellation of these findings suggested urorectal septum malformation. Lung exam was normal with good air entry and clear lung fields, and the infant remained in room air. A chest radiograph revealed normal lung volume and normal expansion, while an abdominal radiograph was remarkable for “double-bubble sign” concerning for duodenal atresia, which was later confirmed by an upper gastrointestinal contrast imaging study. A complete renal ultrasound (Figure 1) was concerning for bilateral renal agenesis which was confirmed by a MAG 3 scan (Figure 2). Bilateral renal agenesis was also noted and confirmed on an MRI (Figure 3). No gonads were identified on abdominal and pelvic ultrasounds. Normal female karyotype was confirmed by chromosome analysis. The infant had rapidly progressing anuric renal failure after birth. In view of complexity of the medical situation and the anticipated need for multiple surgeries on the background of perceived universal fatality of bilateral renal agenesis, a multidisciplinary care conference was held with the family to discuss further direction of care. The medical team supported the family's decision to proceed with full interventions including surgery for duodenal atresia and peritoneal dialysis with goal for renal transplantation in future. She underwent duodenal atresia repair with colostomy and mucous fistula formation. She has remained on room air since birth, and lung development and function were notably normal. She did well on peritoneal dialysis until 4 years of age, and her growth parameters and development remained within normal range. As she was awaiting a renal transplant and needed peritoneal dialysis, her pull through procedure for her colostomy was deferred and was planned to be done at a later time in combination with urogenital reconstruction surgery. She was scheduled for surgery for renal transplantation a few months after her fourth birthday, but she suffered an anaphylactic reaction while anesthesia was being induced, and her surgery was cancelled. She was taken off the transplant list while interdisciplinary teams collaborated to make a plan for safe anesthesia. Unfortunately, a few months later, she was found unresponsive in her bed and was pronounced dead after a full attempt at resuscitation. Notably she was clinically doing well until the time of her demise, and a full autopsy failed to reveal a definite cause of death. All cultures were negative, and it was determined that the most likely cause of death was an electrolyte imbalance that caused cardiac arrest. The autopsy also confirmed the diagnosis of bilateral renal agenesis.

## 3. Discussion

The presence of amniotic fluid (AF) is vital to normal fetal lung development. Renal agenesis is known to result in oligohydramnios or anhydramnios and subsequent sequelae including pulmonary hypoplasia and Potter's sequence. There have been a few case reports of bilateral renal agenesis with normal pulmonary function occurring in monoamniotic discordant twins [2, 3]. In monoamniotic twins, it has been proposed that since the fetuses share a common amniotic cavity, the amniotic fluid volume is maintained by the twin with normal kidneys, allowing the other twin with renal agenesis to develop normal lungs. In these cases, there has been no reported survival beyond 2 months of age. Our patient is the first reported case in a singleton pregnancy, and it is unclear how sufficient amniotic fluid volume had been maintained to support normal lung development.

Normal AF volume is maintained by a dynamic and complex balance between the pathways of amniotic fluid production and amniotic fluid removal. At different stages of gestation, different mechanisms contribute to production and elimination of amniotic fluid. At early stages of gestation, fetal skin is not keratinized, and there is rapid bidirectional diffusion between the fetus and AF [4]. At this stage, the composition of AF is similar to fetal plasma. Fetal urination and fetal swallowing do not significantly contribute to the AF balance until the second trimester. Fetal skin keratinization is complete by around 25 weeks of gestation, and at this point five pathways of AF exchange come into play to maintain normal AF balance [1]. Fetal urine makes the predominant contribution to AF volume, along with contributions from oral, nasal, tracheal, and pulmonary secretions. Major pathway for removal of amniotic fluid is by fetal swallowing. Fluid and solutes also pass from AF to the fetus across an intramembranous pathway. This intramembranous transfer occurs across the amnion, and the amount of fluid transferred in this manner is highly variable. Studies suggest that it is the intramembranous pathway that is primarily responsible for maintaining a normal AF volume balance. A transmembranous pathway by which AF moves across fetal membranes into maternal circulation has a very minimal contribution to AF removal.

There are two possible theories for our patient with bilateral renal agenesis having normal pulmonary function. Our patient had associated duodenal atresia, which would make AF removal by fetal swallowing ineffective, and we suspect this could have contributed to maintaining a somewhat normal AF volume. But, at the same time, it is important to note that only about a third of fetuses with esophageal atresia and two thirds of fetuses with duodenal or proximal jejunal atresia develop polyhydramnios [5], again indicating the major contribution from the intramembranous pathway in regulating AF volume. It is also possible that a vascular accident of the pelvic arterial supply later in pregnancy resulted in the bilateral renal agenesis. This theory is supported by the atresia of several pelvic organs including ovaries, uterus, bladder, and the genital anomalies.

## 4. Conclusion

This child had an unusual presentation in that she presented with normal pulmonary function in the presence of bilateral renal agenesis. This is the first reported case occurring in a singleton pregnancy and the longest documented survival in an infant with bilateral renal agenesis. Most importantly, this case challenges the general consensus of universal fatality associated with bilateral renal agenesis.
